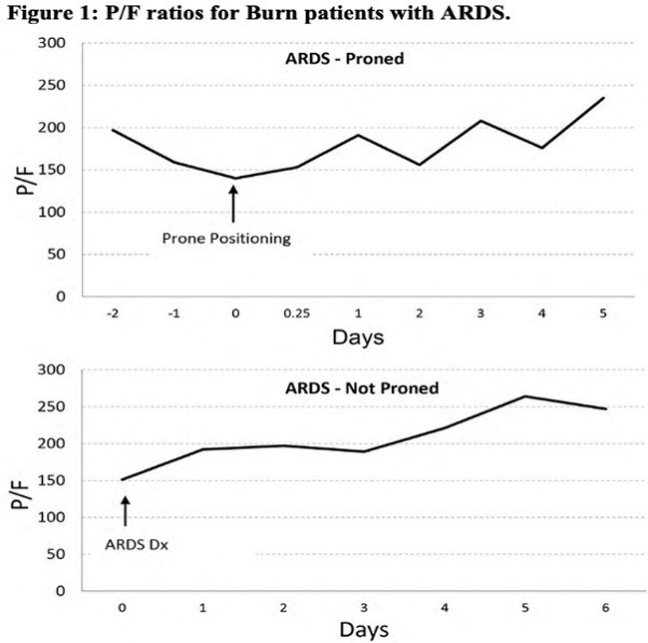# 710 Assessing the Impact of Prone Positioning Among Adult Burn Patients with Acute Respiratory Distress Syndrome

**DOI:** 10.1093/jbcr/irad045.186

**Published:** 2023-05-15

**Authors:** Hannah Nemec, Abigail Cheng, Paul Chestovich, Syed Saquib, Douglas Fraser, Carmen Flores, Joshua Ho, Kavita Batra

**Affiliations:** Kirk Kerkorian School of Medicine at UNLV, Las Vegas, Nevada; University of Nevada Las Vegas, Las Vegas, Nevada; Kirk Kerkorian School of Medicine at UNLV, Las Vegas, Nevada; Kirk Kerkorian School of Medicine at UNLV/UMC Lions Burn Care Center, Las Vegas, Nevada; Kirk Kerkorian School of Medicine at UNLV, Las Vegas, Nevada; Kirk Kerkorian School of Medicine at UNLV, Las Vegas, Nevada; Kirk Kerkorian School of Medicine at UNLV, Las Vegas, Nevada; Kirk Kerkorian School of Medicine at UNLV, Las Vegas, Nevada; Kirk Kerkorian School of Medicine at UNLV, Las Vegas, Nevada; University of Nevada Las Vegas, Las Vegas, Nevada; Kirk Kerkorian School of Medicine at UNLV, Las Vegas, Nevada; Kirk Kerkorian School of Medicine at UNLV/UMC Lions Burn Care Center, Las Vegas, Nevada; Kirk Kerkorian School of Medicine at UNLV, Las Vegas, Nevada; Kirk Kerkorian School of Medicine at UNLV, Las Vegas, Nevada; Kirk Kerkorian School of Medicine at UNLV, Las Vegas, Nevada; Kirk Kerkorian School of Medicine at UNLV, Las Vegas, Nevada; Kirk Kerkorian School of Medicine at UNLV, Las Vegas, Nevada; University of Nevada Las Vegas, Las Vegas, Nevada; Kirk Kerkorian School of Medicine at UNLV, Las Vegas, Nevada; Kirk Kerkorian School of Medicine at UNLV/UMC Lions Burn Care Center, Las Vegas, Nevada; Kirk Kerkorian School of Medicine at UNLV, Las Vegas, Nevada; Kirk Kerkorian School of Medicine at UNLV, Las Vegas, Nevada; Kirk Kerkorian School of Medicine at UNLV, Las Vegas, Nevada; Kirk Kerkorian School of Medicine at UNLV, Las Vegas, Nevada; Kirk Kerkorian School of Medicine at UNLV, Las Vegas, Nevada; University of Nevada Las Vegas, Las Vegas, Nevada; Kirk Kerkorian School of Medicine at UNLV, Las Vegas, Nevada; Kirk Kerkorian School of Medicine at UNLV/UMC Lions Burn Care Center, Las Vegas, Nevada; Kirk Kerkorian School of Medicine at UNLV, Las Vegas, Nevada; Kirk Kerkorian School of Medicine at UNLV, Las Vegas, Nevada; Kirk Kerkorian School of Medicine at UNLV, Las Vegas, Nevada; Kirk Kerkorian School of Medicine at UNLV, Las Vegas, Nevada; Kirk Kerkorian School of Medicine at UNLV, Las Vegas, Nevada; University of Nevada Las Vegas, Las Vegas, Nevada; Kirk Kerkorian School of Medicine at UNLV, Las Vegas, Nevada; Kirk Kerkorian School of Medicine at UNLV/UMC Lions Burn Care Center, Las Vegas, Nevada; Kirk Kerkorian School of Medicine at UNLV, Las Vegas, Nevada; Kirk Kerkorian School of Medicine at UNLV, Las Vegas, Nevada; Kirk Kerkorian School of Medicine at UNLV, Las Vegas, Nevada; Kirk Kerkorian School of Medicine at UNLV, Las Vegas, Nevada; Kirk Kerkorian School of Medicine at UNLV, Las Vegas, Nevada; University of Nevada Las Vegas, Las Vegas, Nevada; Kirk Kerkorian School of Medicine at UNLV, Las Vegas, Nevada; Kirk Kerkorian School of Medicine at UNLV/UMC Lions Burn Care Center, Las Vegas, Nevada; Kirk Kerkorian School of Medicine at UNLV, Las Vegas, Nevada; Kirk Kerkorian School of Medicine at UNLV, Las Vegas, Nevada; Kirk Kerkorian School of Medicine at UNLV, Las Vegas, Nevada; Kirk Kerkorian School of Medicine at UNLV, Las Vegas, Nevada; Kirk Kerkorian School of Medicine at UNLV, Las Vegas, Nevada; University of Nevada Las Vegas, Las Vegas, Nevada; Kirk Kerkorian School of Medicine at UNLV, Las Vegas, Nevada; Kirk Kerkorian School of Medicine at UNLV/UMC Lions Burn Care Center, Las Vegas, Nevada; Kirk Kerkorian School of Medicine at UNLV, Las Vegas, Nevada; Kirk Kerkorian School of Medicine at UNLV, Las Vegas, Nevada; Kirk Kerkorian School of Medicine at UNLV, Las Vegas, Nevada; Kirk Kerkorian School of Medicine at UNLV, Las Vegas, Nevada; Kirk Kerkorian School of Medicine at UNLV, Las Vegas, Nevada; University of Nevada Las Vegas, Las Vegas, Nevada; Kirk Kerkorian School of Medicine at UNLV, Las Vegas, Nevada; Kirk Kerkorian School of Medicine at UNLV/UMC Lions Burn Care Center, Las Vegas, Nevada; Kirk Kerkorian School of Medicine at UNLV, Las Vegas, Nevada; Kirk Kerkorian School of Medicine at UNLV, Las Vegas, Nevada; Kirk Kerkorian School of Medicine at UNLV, Las Vegas, Nevada; Kirk Kerkorian School of Medicine at UNLV, Las Vegas, Nevada

## Abstract

**Introduction:**

Critically-ill burn patients are at high risk for extensive adverse systemic complications due to the severe inflammatory reaction following deep thermal injury. A common but dangerous pulmonary complication associated with deep burns is acute respiratory distress syndrome (ARDS), with incidence and mortality ranging up to 24% and 31%, respectively. Prone positioning (PP) as a means of improving mortality in patients with ARDS has become more widely utilized. However, PP is not well described in the burn population and remains controversial due to the risk of wound complications. We hypothesize that PP in burn patients with ARDS would improve mortality compared to non-prone (NP) patients without adverse outcomes.

**Methods:**

Retrospective data were collected for all burn patients with ARDS from 2018-21. A total of 7 patients who underwent prone positioning (PP) were matched via TBSA, age, and gender 1:2 with 14 patients not proned (NP). Categorical variables were presented as count (percentage) and continuous variables presented as mean (SD). P values were two-sided and P< 0.05 significant.

**Results:**

A total of 58 adult burn patients with ARDS were identified from the burn registry between the years of 2018-2021 and 7 of these were identified as having been treated with PP. Matching was completed to identify 14 NP burn patients with ARDS. Characteristics of these groups are shown in Table 1. The PP patients had significantly higher APACHE II scores, airway pressure release ventilation (APRV) usage, and tracheostomy placement. For the PP patients, mean days to ARDS diagnosis from injury was 4.7±2.6, mean days to PP from ARDS diagnosis was 4.1±6.0, and total days prone was 4.3±3.5. P/F ratios for both the PP and NP groups are shown in Figure 1. P/F ratios for the PP group were declining prior to PP and increased after PP. P/F ratios for the NP group gradually improved without PP. There was no difference in mortality between the two groups (PP=42.8%,NP=57.1%,p=0.6) despite the significantly increased APACHE II scores. No wound complications were noted in the PP group.

**Conclusions:**

ARDS remains a significant cause of mortality in burn patients. The PP patients had similar mortality compared to the NP group despite a two-fold increased expected mortality estimated by the Apache II scores. None of the PP patients had wound complications due to PP. PP appears to be a safe and useful adjunct therapy for burn patients with severe ARDS.

**Applicability of Research to Practice:**

ARDS is a significant cause of mortality in severe burn patients, this study evaluates PP as a treatment.